# Chemokines and their Receptors: Multifaceted Roles in Cancer Progression and Potential Value as Cancer Prognostic Markers

**DOI:** 10.3390/cancers12020287

**Published:** 2020-01-24

**Authors:** Ha Thi Thu Do, Chang Hoon Lee, Jungsook Cho

**Affiliations:** College of Pharmacy, Dongguk University-Seoul, Goyang, Gyeonggi 10326, Korea; doha201191@gmail.com (H.T.T.D.); uatheone@dongguk.edu (C.H.L.)

**Keywords:** cancer, chemokines, prognostic marker, immune cell recruitment, tumor growth and proliferation, angiogenesis, metastasis

## Abstract

Chemokines are chemotactic cytokines that mediate immune cell chemotaxis and lymphoid tissue development. Recent advances have indicated that chemokines and their cognate receptors play critical roles in cancer-related inflammation and cancer progression. On the basis of these findings, the chemokine system has become a new potential drug target for cancer immunotherapy. In this review, we summarize the essential roles of the complex network of chemokines and their receptors in cancer progression. Furthermore, we discuss the potential value of the chemokine system as a cancer prognostic marker. The chemokine system regulates the infiltration of immune cells into the tumor microenvironment, which induces both pro- and anti-immunity and promotes or suppresses tumor growth and proliferation, angiogenesis, and metastasis. Increasing evidence indicates the promising prognostic value of the chemokine system in cancer patients. While CCL2, CXCL10, and CX3CL1/CX3CR1 can serve as favorable or unfavorable prognostic factors depending on the cancer types, CCL14 and XCL1 possess good prognostic value. Other chemokines such as CXCL1, CXCL8, and CXCL12 are poor prognostic markers. Despite vast advances in our understanding of the complex nature of the chemokine system in tumor biology, knowledge about the multifaceted roles of the chemokine system in different types of cancers is still limited. Further studies are necessary to decipher distinct roles within the chemokine system in terms of cancer progression and to validate their potential value in cancer prognosis.

## 1. Introduction

The immune system interacts closely with tumor cells over entire stages of cancer progression from tumor initiation and development to metastasis, facilitating either cancer inhibition or promotion. The balance of these actions determines the eventual outcomes, which, in cases of clinically poor outcomes, are the consequences of immune evasion by tumors [1]. The tumor microenvironment (TME) comprises not only tumor cells, but also immune cells and the surrounding stromal cells. Interestingly, cancer cells can take advantage of these immune cells to help them escape the host’s immune system. In addition, the movement of different subsets of immune cells into the TME is orchestrated by the chemokine system, followed by the regulation of tumor immune responses in a spatiotemporal manner [2,3], and cancer-related inflammation [4]. 

Chemokines are a large family of low-molecular-weight (8 to 14 kDa) chemotactic cytokines [5], which are well-known for their roles in mediating immune cell recruitment [6] and lymphoid tissue development [7]. Chemokines can also directly impact tumor cells and endothelial cells in the TME to regulate tumor cell growth and proliferation, angiogenesis, cancer stem-like cell properties, invasiveness, and metastasis. Hence, chemokines directly and indirectly influence tumor immunity and cancer progression, resulting in a substantial impact on cancer therapy and outcomes [8]. Cancer immunotherapy targeting the chemokine system was recently introduced with several achievements. Mogamulizumab, an anti-CCR4 antibody, was clinically approved for the treatment of relapsed and refractory adult T cell leukemia-lymphoma [9]. Additionally, plerixafor (also known as AMD3100), a CXCR4 antagonist, was approved for the mobilization of hematopoietic stem cells for transplantation in patients with non-Hodgkin’s lymphoma (NHL) or multiple myeloma (MM) [10]. These advances have led to the recognition of chemokines and chemokine receptors as promising targets for cancer immunotherapy, and therefore, in-depth knowledge about the roles and mechanisms of the chemokine system in cancer is crucial for the development of drugs for cancer treatment.

Current standard therapies for most cancers do not benefit all patients. Therefore, the identification of applicable prognostic biomarkers is of great clinical value, not only to improve the therapeutic outcomes but also to provide novel therapeutic targets. Because of its powerful effects on cancer progression, the chemokine system is a potential marker that could predict outcomes for cancer patients. The present review summarizes the essential roles of the complex network of chemokines and their receptors in cancer progression. Furthermore, we discuss the prognostic value of the chemokine system, which has been investigated in diverse cancer types.

## 2. Chemokines and Chemokine Receptors

The chemokine family is divided into four major subfamilies (CC, CXC, CX3C, and C) based on the number and location of the highly conserved cysteine residues at the N-terminus of the chemokine ligands and the presence or absence of intervening amino acids. Whereas the CC, CXC, and CX3C chemokines have zero, one, and three non-conserved amino-acid residues between the first two cysteine residues, respectively, the C chemokines lack the first and third of the four conserved cysteine residues [11,12]. A nomenclature system has been developed, in which the chemokine ligands in the CC, CXC, CX3C, and C subfamilies are named CCL, CXCL, CX3CL, and XCL, respectively. These chemokines are recognized by seven transmembrane-domain G protein-coupled receptors (GPCRs), which are categorized and named CCR, CXCR, CX3CR, and XCR, respectively, based on their chemokine ligand sources [11,12]. Notably, some chemokines are ligands of more than one GPCR, and conversely, some GPCRs bind to more than one type of chemokines, inducing diverse effects. The chemokine receptors and their ligand pairings known in humans and mice are listed in Table 1.

As described above, chemokines function as chemoattractants, orchestrating the infiltration of immune cells to the TME, influencing tumor cell growth and proliferation, angiogenesis, and metastasis, and therefore contributing to cancer initiation and development [16]. These multifaceted roles of chemokines and their receptors in cancer progression are discussed in the following section.

## 3. Roles of Chemokine System in Cancer Progression 

A number of studies have demonstrated the roles of chemokines and chemokine receptors in cancer progression. The recent advances in our understanding of the various chemokine systems and their differential roles in the recruitments of key immune cells, tumor cell growth and proliferation, angiogenesis, and tumor metastasis are discussed.

### 3.1. Roles of Chemokine System in Immune Cell Recruitment 

Chemokine receptors are expressed in various kinds of immune cells (Table 1). The trafficking of these cells is directed by chemokine gradients that guide the cells to migrate to locations with high concentrations of chemokines [16], inducing either pro- or antitumor immune responses in the primary tumors and metastatic sites [8]. We discuss the roles of chemokines and their receptor networks which control the recruitment of key immune cells into the TME and demonstrate how the infiltrated cells regulate the immune response and tumor development.

#### 3.1.1. T Cells

T cells are leukocytes expressing T cell receptors (TCRs) that recognize antigens presented by the major histocompatibility complex (MHC). T cells are classically divided into CD8^+^ cytotoxic T lymphocytes (CTLs) and CD4^+^ T cells, which recognize peptides presented by MHC class I and MHC class II, respectively [17]. CD4^+^ T cells include several T helper (T_h_) cells, among which T_h_1 and T_h_2 are the most important, and other T cell types such as T_reg_.

CD8^+^ CTLs are considered critical mediators of the antitumor response [17]. As shown in Table 1, various chemokine receptors, including CCR4/5, CXCR3, and CX3CR1, are expressed on CD8^+^ CTLs. Thus, their corresponding ligands such as CCL5, CXCL9, CXCL10, and CX3CL1 effectively guide CD8^+^ CTL mobilization from regional lymph nodes to tumor tissues [18]. CD8^+^ CTLs have powerful cytotoxic abilities due to the secretion of effector cytokines or cytotoxic molecules such as perforin and granzyme B, or interactions of the CD95 (Fas) molecule and its ligand CD95L, ultimately resulting in apoptosis in tumor cells [19,20,21]. Due to these antitumor effects, CD8^+^ CTL expression was reported to be associated with a favorable prognosis in breast cancer (BC) patients [22].

T_h_1 cells also have potent antitumor effects in the TME. Chemokines such as CXCL9 and CXCL10 can orchestrate the migration of effector CXCR3^+^ immune cells such as T_h_1 cells into the tumor sites, subsequently shaping both the tumor immunity and therapeutic responses [23,24,25,26]. Importantly, interferon gamma (IFN-γ) produced by T_h_1 cells not only has direct effects on arresting cellular proliferation, promoting apoptosis, and reducing angiogenesis but also on improving CTL responses to robust antitumor immunity [17]. Notably, T_h_1 immune responses in lymph nodes signify a protective effect in colon cancer patients [27] and can be considered a marker for prolonged survival in pancreatic ductal adenocarcinoma (PDAC) patients [28]. 

By contrast with T_h_1 cells, T_h_2 cells have protumor functions. Interleukin (IL)-4 is the signature cytokine for T_h_2 cells and functions as either an inducer or an effector cytokine of the cells [29]. The chemokines CCL8, CCL17, and CCL22 have chemoattractions with T_h_2 cells expressing CCR8 and CCR4 (Table 1) [25,30,31]. T_h_2 lymphocytes help B cells produce antibodies and suppress the action of cytotoxic T cells [16]. Intriguingly, a low circulating level of IL-4 can identify resectable pancreatic adenocarcinoma patients with better prognosis [32]. 

T_reg_ cells play an essential role in maintaining self-tolerance and immune homeostasis [33]. The recruitment of T_reg_ cells to TME is mediated by chemotaxis of CCL20/CCR6 [34], CCL22/CCR4 [35], CCL28/CCR10 [36], and CXCL12/CXCR4 [37,38]. Importantly, T_reg_ cells abate tumor-specific T cell immunity involving CTLs, T_h_, natural killer (NK), and natural killer T cells, contributing to tumor growth and metastasis [33,35,39]. In addition, the cells can promote inflammation in the TME *via* expressing inflammatory cytokines [40]. By providing an escape route for cancers from the immune response, the expression of T_reg_ cells is significantly correlated with worse overall survival (OS) in the majority of solid tumors. However, it is associated with better survival in several cancers, including colorectal, head and neck, or esophageal cancers with unclear mechanisms [41].

#### 3.1.2. Natural Killer Cells

NK cells are well-known to play a role in antitumor immune responses. Their migration to inflamed tissues including tumor sites involves a series of chemokine receptors such as CCL3-5/CCR5 [42], CXCL10/CXCR3 [43], and CX3CL1/CX3CR1 [44]. Similar to CTLs, the cell-mediated cytotoxicity of NK cells also occurs *via* effector cytokines, cytotoxic molecules, and the Fas pathway [19,20,21,45]. Moreover, the elimination of tumors mediated by NK cells, subsequently, leads to tumor-specific T cell responses [45]. Especially, a high infiltration density of NK cells in a tumor nest is associated with better OS in esophageal cancer [46]. 

#### 3.1.3. B Cells

B cells are central players in humoral immunity due to their antibody production capacity [47]. Chemokine axes such as CCL19, CCL21/CCR7, CCL20/CCR6, CXCL12/CXCR4, and CXCL13/CXCR5 (Table 1) correlate with B cell infiltration to tumor sites [15,48]. B cells exhibit antitumor functionality by directly killing tumor cells, producing specific antibodies for tumor antigens, acting as antigen-presenting cells (APCs) for T cell activation and memory T cell development, and facilitating CD4^+^ and CD8^+^ T cell immune responses [49,50,51,52,53]. However, B cells induce protumor effects by activating STAT3, promoting tumor angiogenesis and facilitating tumor progression [54]. Due to the dual roles of B cells, their high density is associated with good outcomes in non-small cell lung cancer (NSCLC) [55] but poor outcomes in ovarian cancer [56,57].

#### 3.1.4. Dendritic Cells (DCs)

DCs have opposite effects in tumor response depending on their maturation stage. Immature DCs (iDCs) present antigens to T cells, and then induce immune tolerance including the generation of inducible T_reg_ cells, T cell anergy and deletion [58]. iDCs are attracted to tumor tissue sites through CCL20, CXCL12, and CXCL14 chemotaxis [59,60,61,62]. Conversely, mature DCs (mDCs) assist the priming of CD4^+^ T_h_ cells and CD8^+^ CTLs, the activation of B cells, and the initiation of adaptive immune responses [58]. CCL19 attracts mDCs and lymphocytes expressing CCR7 in T cell-rich areas, and thereby helping DCs meet tumor-associated antigen-specific T cells [63]. Due to their capacity to mediate T cell immunity, DCs can be used as adjuvants for cancer vaccination [58]. 

#### 3.1.5. Neutrophils

Neutrophils also have a crucial regulatory role in tumor establishment and development [64]. Chemokines such as CCL2, CCL3, CXCL1, CXCL2, CXCL5, CXCL8, and CXCL12 promote neutrophil infiltration to tumors [64]. Importantly, neutrophils induce antitumor functions through direct cytotoxicity, antibody-dependent cellular cytotoxicity, and specific antigen presentation [65]. Nevertheless, neutrophils can induce genotoxicity and promote excessive angiogenesis and tumor proliferation [65]. Additionally, neutrophils can facilitate tumor metastasis by forming premetastatic niches and neutrophil extracellular traps (NETs) [14,64,65,66,67]. Intriguingly, since neutrophils have both pro- and antitumor effects, a higher density of neutrophils is associated with better response to 5-fluorouracil-based therapy in CRC patients [68], but worse clinical outcomes in the other types of human cancers [69].

#### 3.1.6. Macrophages 

Macrophages are attracted to tumor sites expressing chemotactic factors such as CCL2, CCL5, CCL7, CCL8, CXCL1, and CXCL12 (Table 1) [18,70]. Macrophages have been classified as classical M1 (antitumor macrophages) and alternative M2 (protumor macrophages) polarized subtypes. M1 macrophages can directly kill tumor cells and produce proinflammatory cytokines [71]. Contrarily, tumor-associated macrophages (TAMs) possess the properties of M2-polarized macrophages, produce immunosuppressive molecules to promote T_reg_ cells, and suppress antitumor immunity [18,71,72,73]. Indeed, TAMs produce growth factors such as vascular endothelial growth factor (VEGF), fibroblast growth factor (FGF), and prostaglandin to support angiogenesis and tumor growth [18]. TAMs establish a niche for cancer stem cells (CSCs) and support the epithelial–mesenchymal transition (EMT), which leads to cell migration and metastasis [73]. Collectively, macrophages exhibit either anti- or protumor functions based on their classification (M1 or M2).

#### 3.1.7. Myeloid-Derived Suppressor Cells (MDSCs)

Tumor tissues contain MDSCs, which can suppress innate and adaptive antitumor immunity and contribute to tumor progression [74,75]. The infiltration of MDSCs into tumors is related to numerous chemokine and receptor axes such as CCL2, CCL7, CCL8/CCR2, CCL5/CCR5, CXCL1, CXCL2, CXCL5/CXCR2, and CXCL12/CXCR4 [18]. MDSCs that migrate to tumor sites increase STAT1 activity, leading to low levels of reactive oxygen species (ROS) and high levels of iNOS, NO, and arginase-1, which inhibit CD8^+^ T cell functions in a nonspecific manner [72]. Moreover, MDSCs can endow cancer cells with stem cell-like properties and are linked with cancer stemness [8]. MDSCs also support tumor angiogenesis by producing angiogenic factors such as VEGF, platelet-derived growth factor (PDGF), transforming growth factor beta, CXCL8, and matrix metalloproteinases (MMPs) such as MMP-2 and MMP-9 [18]. Data from one meta-analysis in solid tumors demonstrated that elevated levels of circulating MDSCs are negatively associated with the survival outcomes in most cancers [76].

Taken together, the chemokine system plays key roles in regulating the infiltration of immune cells into the TME, which leads to diverse functions in tumor immunity. While CD8^+^ CTLs, T_h_1, and NK cells induce antitumor responses, T_h_2, T_reg_ cells, and MDSCs stimulate protumor functions, and B cells, DCs, neutrophils, and macrophages probably exhibit both anti- and protumor effects. Interestingly, however, one chemokine axis can attract different kinds of immune cells, which generate contrasting effects. The functional redundancies not only cause difficulties in the development of anticancer drugs that target the chemokine system but also lead to opposite prognoses. For example, the CCL5/CCR5 axis, which can recruit CD4^+^ and CD8^+^ T cells [77] and NK cells [42], can predict the improved efficiency of DC-immunotherapy in NSCLC [78]. Nevertheless, this pair also exhibits chemoattraction to TAMs and MDSCs [79,80], and has been reported to correlate with poor outcomes in BC patients [81].

### 3.2. Roles of Chemokines in Tumor Growth and Proliferation

Whereas normal cells strictly control the cellular homeostasis by regulating the synthesis and release of growth factors, tumor cells disrupt the regulatory mechanisms of the host for growth factor production and, then, sustain their growth and proliferative signals [82]. Numerous studies have demonstrated that the chemokine system is involved in tumor growth and proliferation through several mechanisms.

One of the mechanisms by which some chemokines such as CCL2 or CXCL8 promote tumor growth and proliferation involves acting as autocrine or paracrine growth factors [83,84,85]. Furthermore, chemokines including CCL2 and CCL5 contribute to tumor proliferation through the formation of an immunosuppressive TME by recruiting T_reg_ cells or inflammatory monocytes and macrophages [86,87,88]. 

Phosphoinositide 3-kinase (PI3K)/AKT and extracellular signal-regulated protein kinases 1 and 2 (ERK 1/2) pathways are two key cellular signalling involved in tumor cell survival and proliferation [89,90]. Interestingly, these pathways are utilized by a series of chemokines and their receptors, such as CCL20/CCR6 [91], CCL25/CCR9 [92], CXCL1/CXCR2 [93], CXCL8/CXCR1-2 [94], CXCL12/CXCR4 [95], and CX3CL1/CX3CR1 [96], to inhibit apoptosis and promote tumor cell growth and proliferation. Intriguingly, both the PI3K/AKT and ERK 1/2 pathways induced by interactions between chemokines and their receptors can lead to nuclear factor kappa B (NF-κB) activation [97]. While the NF-κB pathway induces the upregulated expression of some chemokines such as CCL2, CCL5, CXCL5, CXCL8, CXCL10, CXCL12, and CX3CL1, it also participates in the antiapoptotic and proliferative effects of CCL5, CCL20, CXCL8, and CXCL12 in pancreatic cancer [98]. Importantly, chemokines can promote tumor cell survival by regulating the balance between NF-κB-associated pro- and antiapoptosis proteins. CCR5 and CX3CL1 were demonstrated to promote the expression of antiapoptotic and tumor-promoting proteins such as Bcl-xl, Bcl-2, and C-IAP1, as well as to reduce the expression of apoptotic proteins including cleaved caspase-3 and -9, PARP, and Bax *via* the NF-κB pathway [99,100]. 

In contrast, chemokines also inhibit tumor growth and proliferation. The term “cellular senescence” has been used to describe a state of stable and long-term proliferative arrest, despite maintained viability and metabolic activities [101]. Oncogene-induced senescence (OIS) is a highly stable antiproliferative response to oncogenic stress and acts as an effective barrier to tumor progression [102,103]. In the early stages of tumorigenesis, chemokines such as the CXCL1/CXCR2 axis can mediate OIS through NF-κB signalling to restrict tumor growth. However, in late stages of tumorigenesis, cellular senescence becomes a source of inflammation, recruiting MDSCs into the inflamed tumor, generating an immune suppressive microenvironment, and allowing cancer cell growth [103,104]. Moreover, CCL14 attenuates hepatocellular carcinoma (HCC) cell proliferation by inhibiting cell cycle progression and promoting apoptosis *in vitro* and suppresses HCC growth *in vivo via* the Wnt/β-catenin signalling pathway [105]. 

Taken together, the functions of chemokines and their receptors in inducing either pro- or antitumorigenic activities are highly complicated. On the one hand, chemokine systems can promote tumor growth and proliferation through the autocrine growth factor function, generation of immunosuppressive TME, and the PI3K/AKT and NF-κB signalling pathways. On the other hand, they can induce OIS or the Wnt/β-catenin signalling pathway to mitigate tumor development. Hence, future studies should be more focused on elucidating the underlying action mechanisms for these chemokine systems to decipher their distinct roles in tumor biology and to discover new targeted therapies for effective cancer treatment.

### 3.3. Roles of Chemokine System in Tumor Angiogenesis

Like normal tissues, tumors require sustenance from nutrients, oxygen, and the ability to excrete metabolic wastes and carbon dioxide, which are addressed by the process of angiogenesis, a tumor-associated neovasculature [82]. Many chemokine systems have been found to play important roles in tumor angiogenesis [106].

CXC chemokines, depending on the expression of the glutamic-leucine-arginine (ELR) motif at the N-terminal, can be classified into ELR^+^ chemokines with angiogenic effects and ELR^−^ chemokines with angiostatic effects [107]. Angiogenic ELR^+^ CXC chemokines comprise CXCL1, CXCL2, CXCL3, CXCL5, CXCL6, CXCL7, and CXCL8. Chemokines such as CXCL6 and CXCL8 can specifically bind and activate both CXCR1 and CXCR2 (Table 1), whereas other angiogenic ELR^+^ CXC chemokines mediate their angiogenic activity through CXCR2 [108,109]. Although CXCL12 is one of the ELR^−^ CXC chemokines, it had been exceptionally implicated as a strong angiogenic chemokine [108]. All the angiogenic CXC chemokines contain a putative cis-element that recognizes NF-κB, leading to tumor-associated angiogenesis [109]. Furthermore, by working alone or interacting with other angiogenic factors such as VEGF, basic FGF (bFGF), and prostacyclin, the CXC axes such as CXCL8/CXCR2 and CXCL12/CXCR4 can act in either a direct, parallel, or serial manner to stimulate angiogenesis [109,110,111]. Interestingly, the pro-angiogenic effect of VEGF and CXCL8 was demonstrated to be further associated with the activation of neutrophils [112]. Furthermore, other chemokines such as CCL1, CCL2, CCL3, CCL11, CCL15, CCL16, CCL23, and CX3CL1 have also been implicated in tumor neovascularization by promoting migration and differentiation with or without the proliferation of endothelial cells and inducing new blood vessel formation [113,114,115,116,117,118,119,120]. CCL2 can recruit angiogenic factor-producing leukocytes such as macrophages into the TME to accelerate angiogenesis [121]. 

Except for CXCL12, other ELR^−^ members of the CXC chemokine family including CXCL4, CXCL4L1, CXCL9, CXCL10, CXCL11, and CXCL14 are angiostatic [108,109]. CXCL4L1 is produced by the nonallelic variant gene of CXCL4 and differs from CXCL4 in only three amino acid residues but has more potent angiostatic effect than CXCL4 [109]. CXCR3 is a major receptor that has been identified for angiostatic CXC chemokines including CXCL4, CXCL9, CXCL10, and CXCL11. These chemokines are involved in the recruitment of T_h_1 cells expressing CXCR3, which acts as a receptor for the inhibition of angiogenesis [108,109,122]. Interestingly, the angiostatic capabilities of CXCL4 and CXCL10 also come from their suppression of bFGF and VEGF-induced angiogenesis and their inhibition of endothelial cell proliferation and chemotaxis [108,123,124,125]. CXCL14 has been shown to be a potent angiogenesis inhibitor but its receptor and underlying action mechanism remain unidentified [65,109]. Furthermore, CCL5 binding to CCR5 has also been demonstrated to mediate anti-angiogenic activity with an undefined mechanism [126]. 

In summary, chemokines stimulate or inhibit angiogenesis by the promotion or suppression of angiogenic factors such as VEGF and bFGF and the migration and proliferation of endothelial cells. Another way of chemokines to augment angiogenesis is through the recruitment of immune cells that support angiogenesis to the TME.

### 3.4. Roles of Chemokine System in Tumor Metastasis

Tumor metastasis is the movement of tumor cells from a primary site to progressively colonize distant organs and is a major contributor to the death of cancer patients [127]. After growing and proliferating at the primary tumor site, tumor cells migrate and invade the surrounding extracellular matrix (ECM), then proceed to enter the bloodstream or lymphatic system, becoming circulating tumor cells (CTCs). CTCs are disseminated along chemotactic gradients and induce extravasation at non-random and organ-specific sites, followed by tumor growth and proliferation at the new sites [14]. There are some organs in the body that are more susceptible to tumor metastasis such as the lung, brain, liver, lymph nodes, and bone marrow while others like the skin, kidneys, and pancreas are less prone [128]. While the mammalian body has a variety of active cellular highways, chemokines are considered to act as the “traffic directors” responsible for guiding cells that express appropriate receptors to specific locations. Metastatic cancer cells can “hijack” the chemokine receptor-regulated cell migration highway to initiate metastasis at distant sites [128]. 

At the new distant locations, cancer cells can exploit the chemokine system to establish immune system suppression and angiogenesis for the formation of a pre-metastatic niche, and to facilitate the proliferation of metastatic cancer cells [129]. For example, CCL2 stimulates metastatic seeding of BC cells by increasing the retention of metastasis-associated macrophages [130]. Furthermore, CXCR2 has a key role in metastatic progression, involving the migration of myeloid lineage cells such as neutrophils, macrophages, and MDSCs to both primary tumors and metastases [131]. Interestingly, in BC, the CCL5 secreted by lymphatic endothelial cells within the lungs and lymph nodes directs tumor dissemination into these tissues and promotes metastasis [132].

Cell migration is an integrated multistep process initiated by the process of membrane protrusion, which is driven by localized polymerization of actin filaments on the submembrane [133]. The binding of chemokines to their GPCRs activates a series of downstream signalling pathways that regulate integrin activation (adhesion) and actin cytoskeleton reorganization. This leads to actin polymerization and F-actin formation, followed by pseudopodia formation and, eventually, more efficient migration and invasion of tumor cells [134,135]. The CCL5/CCR5, CCL21/CCR7, and CXCL12/CXCR4 axes have been shown to promote cell migration through this mechanism [136,137]. 

The EMT is a phenotypic change from polarized epithelial cells to mesenchymal cells, resulting in the loss of cell-cell adhesion and cell polarity, increased migratory capacity and invasiveness, enhanced resistance to apoptosis, and substantial promotion of the production of ECM components [138,139]. Two of the most important properties that promote metastasis, namely invasiveness and stemness, converge during EMT [140]. Interestingly, various chemokines have been implicated to contribute to EMT progression in cancer cells. EMT can be induced by CXCL8 and its receptors through overexpression of the transcription factor Brachyury [141], CCL2 with the enhancement of Snail expression [142], the CXCL6/CXCR1/2 axis *via* the PI3K/AKT and Wnt/β-catenin pathways [143], and the CXCL1/LCN2 paracrine axis with the activation of Src signalling [144]. In addition, NF-κB is associated with the EMT induced by CCL5 and CCL18 [145,146]. In contrast, CCL28 treatment has been demonstrated to inhibit cell invasion and EMT in oral squamous cell carcinoma cells [147]. 

CSCs refer to undifferentiated and self-renewing tumor cells, which have the ability to initiate heterogeneous tumors and repopulate metastatic outgrowths [140]. Many studies have demonstrated a correlation between the chemokine system and CSC-like properties in cancer cells. The CXCL12/CXCR4 axis has been well-documented to have various roles in CSCs. The overexpression of CXCR4 or CXCL12γ, an isoform of CXCL12 found in CD44^+^/CD133^+^ prostate CSCs, suggests that one mechanism by which the CXCR4/CXCL12 axis promotes metastasis in prostate cancer is the maintenance of stemness in CSCs [148,149]. In addition, CXCR1 blockage reduced CSC properties in clear cell renal cell carcinoma [150], depleted the CSC population, and reduced systemic metastasis development in BC cells [151]. CCR5^+^ BC cells demonstrated several specific features of CSCs, including increased mammosphere formation, enhanced ability to initiate tumors, and metastatic capacity, as well as improved DNA repair activity [152].

**Figure 1:**
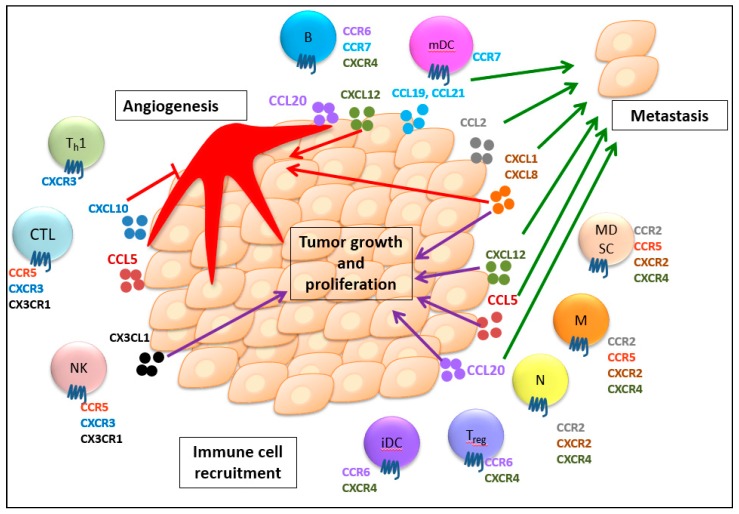
Multifaceted roles of chemokines and their receptors in immune cell recruitment, tumor growth and proliferation, angiogenesis, and metastasis. Chemokines guide the trafficking of different immune cells expressing their respective receptors into the tumor microenvironment, which induces both anti- and protumor immunity. Additionally, the chemokine system generally stimulates tumor growth and proliferation. Chemokines can also regulate angiogenesis with their angiogenic or angiostatic functions. Furthermore, chemokines are involved in tumor migration to secondary sites to develop metastasis. CTL, CD8^+^ cytotoxic T lymphocyte; T_h_1, T helper cell; NK, natural killer cell; T_reg_, regulatory T cell; B, B cell; iDC, immature dendritic cells; mDC, mature dendritic cell; N, neutrophil, M, macrophage; MDSC, myeloid-derived suppressor cell. The purple arrows show the promotion of tumor growth and proliferation. The red arrows indicate the angiogenic effect. The red T line indicates the angiostatic effect. The green arrows indicate the promotion of metastasis. (For detailed information, please see the text.).

Briefly, chemokines promote tumor metastasis through their hijacked cell migration highway, the establishment of a premetastatic niche, formation of pseudopodia, and induction of EMT and CSC properties. Therefore, chemokines that stimulate tumor metastasis can potentially serve as poor prognostic markers for cancer patients. Taken together, the chemokine system plays pivotal roles in regulating immune cell recruitment to the TME, tumor growth and proliferation, angiogenesis, and metastasis. The representative chemokines and chemokine receptors associated with their multifaceted roles in cancer progression are delineated in Figure 1.

## 4. Role of Chemokine System in Cancer Prognosis

So far, this review has described the complicated and multifaceted roles of the chemokine system in cancer progression. These critical roles of the chemokine system could have value in predicting OS in cancer patients. We used the PubMed database as the primary source and Google as the secondary source and searched for relevant articles on the role of chemokine system in cancer prognosis, published up to October 2019. The following key words were employed in the search: chemokine/s, prognosis, prognostic, and cancer. There is increasing evidence from retrospective, prospective, prospective-retrospective studies, which are designed as retrospective analysis of archived tissues prospectively collected with follow-up data, and even meta-analysis studies to demonstrate the potential predictive value of the chemokine system for patients with different kinds of cancers. In this section, we will discuss the roles of chemokines as prognostic factors for cancer patients in correlation with their roles in disease progression.

### 4.1. CCL2

Interestingly, a hypothesis suggested that tumor cells produce CCL2 to amplify the recruitment of monocytes or macrophages, which might kill tumor cells by producing pro-inflammatory cytokines [153]. Even though CCL2 exhibits angiogenic effects *in vivo*, the activity is only observed at certain doses but not at higher doses of CCL2 [154]. Nevertheless, there is a series of results suggesting that the CCL2-dependent signalling pathway could promote the survival of tumor cells [155,156] and stimulate metastasis [157,158,159] and angiogenesis [121]. The dual roles of the CCL2/CCR2 axis in cancer development can lead to opposite results, as patients with higher CCL2 or CCR2 expression had significantly better OS in NSCLC [153] but shorter OS and progression-free survival in diffuse large B cell lymphoma (DLBCL) [160], although evidence about the roles of CCL2/CCR2 in DLBCL is limited. Further understandings are needed to clarify the value of CCL2/CCR2 as a prognostic factor in many different cancer types.

### 4.2. CCL5

The CCL5/CCR5 axis has been demonstrated to promote cancer cell migration through the recruitment and modulation of inflammatory cell activities, followed by the generation of an immunosuppressive environment including TAMs and MDSCs in BC [79,80,161]. Data from a study on BC patients showed that patients with higher serum levels of CCL5 had a greater probability of lymph node metastasis [81]. Similarly, in stage II BC patients, the positive tumor expression of CCL5 alone or when combined with the absence of estrogen receptor-α significantly increased the risk for disease progression [162]. Therefore, CCL5 can be considered a poor prognostic factor for BC, primarily in stage II patients.

### 4.3. CCL14

The functions of CCL14 in cancer progression have not been clearly identified. CCL14 promotes apoptosis, alleviates HCC cell proliferation and growth by inhibiting cell cycle progression through the Wnt/β-catenin signalling pathway, and contributes to longer OS in HCC patients [105]. Consistently, high expression of CCL14 genes effectively improved survival times in HCC [163]. In contrast, however, CCL14 was reported to promote bone marrow infiltration, proliferation, and the polarization of macrophages, which was considered to be associated with chemoresistance in MM [164]. More studies are needed to discover the mechanisms of action for this chemokine in cancer development and its prognostic functions in HCC and other cancer types. 

### 4.4. CCL20

Studies have reported that the CCL20/CCR6 axis plays a key role in the tumor-chemokine network and promotes tumor progression in HCC and CRC. This axis has been shown to stimulate cell proliferation, probably *via* regulating the expression of p21, p27, and cyclin-D1 [91,165], induce EMT *via* PI3K/AKT and Wnt/β-catenin pathways [166], and eventually, promote metastasis [167,168]. The CCL20/CCR6 network facilitates T_reg_ activity and induces immune suppression to mediate cancer cell elimination and metastasis [34]. With these protumor effects, CCL20 expression in HCC [169] and the co-expression of CCL20 and CXCL8 in CRC [167] were negatively associated with patient outcomes.

### 4.5. CCR7

CCR7 has been well-documented to comprehensively promote tumor development in many cancer types. CCR7 can weaken the host’s antitumor immunity by downregulating IFN-γ mediated inflammation in melanoma [170]. In addition, CCR7 has been demonstrated to inhibit apoptosis and stimulate proliferation by promoting G2/M phase progression through the ERK1/2 pathway in NSCLC [171]. CCR7 also induces tumor angiogenesis by promoting VEGF-C expression in prostate cancer [172]. Moreover, it enhances the proliferation and migration of endothelial cells and increases angiogenic capacity *via* the NF-κB/VEGF pathway in esophageal squamous carcinoma cells (ESCC) [173]. CCR7 has also been found to induce EMT in PDAC and lung cancer (LC) [174,175], promote MMP-2 and -9 expression in bladder cancer [176], and facilitate tumor cell dissemination, migration, and eventually metastasis formation [177]. With the strong protumor functions, the expression of CCR7 or CCL21 has been reported to be strongly correlated with poor survival [169,178]. Hence, CCR7 could become a predictive marker for a number of cancers, including colorectal liver metastasis.

### 4.6. CXCL1

CXCL1 has been reported to play an important role in CRC progression and metastasis by inducing glycolysis [179]. CXCL1 is also an ELR^+^ CXC chemokine that induces angiogenesis by binding to CXCR2 [109]. In addition, CXCL1 produced by TAMs recruits CXCR2^+^ MDSCs for the pre-metastatic niche to stimulate liver metastases in CRC [70]. Moreover, CXCL1 directly represses T cell infiltration and mitigates sensitivity to immunotherapy in pancreatic cancer [180]. Consistently, due to the tumor-promoting effects, high CXCL1 expression has been shown to be a risk factor for cancer prognosis, with poor OS, advanced tumor, node, and metastasis stage, and lymph node metastasis. This result strongly suggests the prognosis value of CXCL1 for various cancers including CRC, pancreatic cancer, and others [181]. It has also been suggested that CXCL1 mediates radioresistance by regulating the DNA damage response in a ROS-dependent manner in ESCC [182].

### 4.7. CXCL8

CXCL8 has been extensively investigated for its functions in promoting tumorigenesis. CXCL8 can stimulate proliferation and survival *via* autocrine activation in CRC [183], cervical cancer [184], and LC [185], or *via* the ERK1/2 pathway in NSCLC [94]. CXCL8 also promotes angiogenesis and cell migration, induces EMT in CRC [183,186], attracts and activates MDSCs to form NETs, and helps tumors evade the immune system [187]. Due to the above protumor effects, high expression of CXCL8 potentially serves as an unfavorable prognostic marker in numerous human cancers, including CRC [167,188], cervical cancer [189], and lung adenocarcinoma [190]. Consistently, CXCL8 has also been a strong predictor for poor outcome in various treatments including chemo-immunotherapy [191] in pancreatic cancer or aflibercept therapy in metastatic CRC [192].

### 4.8. CXCL10

CXCL10 has opposing effects on TME. On the one hand, local production of CXCL10 attracts CTLs into ESCC tissue and probably plays a positive role in patient survival [193]. On the other hand, an *in vitro* study showed that monocytes, orchestrated by CXCL10, promote the migration and invasion of tumor cells in B cell precursor acute lymphoblastic leukemia (ALL), and then stimulate metastasis [194]. Because of the above dual functions, elevated expression of CXCL10 has been associated with favorable outcomes in ESCC [195], but unfavorable prognosis in DLBCL [196].

### 4.9. CXCL12/CXCR4

CXCL12/CXCR4 is a well-investigated axis that is strongly involved in all stages of tumor progression in many kinds of cancer. CXCL12/CXCR4 plays an important role in the recruitment of T_reg_ cells into the TME, and contributes to immune suppressive activities and tumor-related inflammation in HCC and malignant pleural mesothelioma [37,38]. As mentioned above, the CXCL12/CXCR4 pair promotes tumor cell growth and the proliferation of glioblastoma cells *via* the ERK1/2 and AKT pathways [95]. In addition, the axis is involved in tumor angiogenesis *via* VEGF-dependent mechanisms in BC [197] and glioblastoma [198]. This axis also promotes metastasis mediated by actin polymerization, pseudopodia formation [199], and EMT induction [200]. Moreover, CXCR4 plays a key role in the self-renewal of CSCs in drug-resistant NSCLC [201]. Due to the powerful protumor activities of CXCL12/CXCR4 axis, numerous studies have consistently shown that their expression is associated with poor prognosis for patients in esophageal cancer [202], acute myelogenous leukemia [203], BC [204,205], HCC [206], and NSCLC [207]. In addition, CXCR4 expression correlates with the degree of tumor infiltration and promotes a more aggressive phenotype in papillary thyroid carcinoma [208], and is also a negative prognostic marker for response to chemotherapy in NHL [209].

### 4.10. CX3CL1/CX3CR1

The CX3CL1/CX3CR1 axis has both tumor-promoting and suppressive effects in cancer progression, resulting in either favorable or unfavorable prognosis depending on the cancer types. Increased expression of CX3CL1 has been correlated with a better prognosis, with enhanced recruitment of CD8^+^ T and NK cells in gastric adenocarcinoma patients [210]. Furthermore, in HCC patients, CX3CL1/CX3CR1 regulates the cancer cell cycle and tumor progression *via* the autocrine or paracrine systems, which is associated with positive outcomes [211]. In contrast, CX3CL1/CX3CR1 has been proven to induce tumor growth, proliferation, and apoptosis resistance through activating the AKT/NF-κB [100] and JAK/STAT signalling pathways in PDAC [212]. Therefore, a study in PDAC patients showed evidence that high expression of the CX3CL1/CX3CR1 axis in tumor tissues led to a poor prognosis for OS [213].

### 4.11. XCL1

XCL1 attracts CD4^+^ and CD8^+^ T cells, neutrophils [214], and NK cells [215]. It probably augments antitumor responses, and therefore could be crucial in gene transfer immunotherapies in some cancers [214]. Interestingly, higher serum XCL1 levels at diagnosis and their progressive decline during chemotherapy were associated with higher survival in ALL [216]. This could be explained by the progressive decrease of the leukemic burden in the tumor’s response to cancer treatments. However, this result should be taken into consideration because patients with good predictive factors were also younger and had lower white blood cell counts [216]. The mechanism of XCL1 in cancer progression and its role in cancer prognosis should be examined further in future investigations.

In brief, many chemokines and chemokine receptors can act as prognostic markers for cancer patients. CCL2, CXCL10, and CX3CL1/CX3CR1 can serve as both favorable and unfavorable prognostic factors for cancers depending on cancer types. While CCL14 and XCL1 have shown only good prognostic value, other chemokines such as CCL5, CCL20/CCR6, CCR7, CXCL1, CXCL8, and CXCL12/CXCR4 have consistently played the role of poor prognostic markers in different kinds of cancer. The favorable and unfavorable roles of chemokines as prognostic factors in different types of cancers are summarized in Table 2. Interestingly, high levels of multiple chemokines were reported to be strongly associated with worse patient OS in several clinical trials [217,218,219]. In order to identify and validate multiple chemokines as promising and predictive tumor-based biomarkers for patient outcomes, further investigations in various clinical trials of anticancer treatments are necessary.

## 5. Conclusions

It is undeniable that during the past several decades, there have been enormous advances in our knowledge regarding the functions of the chemokine system in cancer. Accumulating evidence strongly supports the multifaceted roles of chemokines and their receptors in tumor progression. Importantly, chemokines act as chemoattractants to recruit both anti- and protumor immune cells into the TME. Furthermore, most chemokines function as promoting factors for tumor growth and proliferation, angiogenesis, and metastasis; however, some other chemokines have the opposite effects. The complicated effects of the chemokine system in cancer could be due to the promiscuity of chemokine and chemokine receptor interactions. One chemokine-receptor pair can serve as tumor suppressors in one type of cancer and as tumor promoters in other types of cancer. Therefore, some chemokines such as CCL2, CXCL10, and CX3CL1/CX3CR1 can be either favorable or unfavorable cancer prognostic factors depending on the cancer types. In contrast, CCL14 and XCL1 only serve as good prognostic factors for cancer patient outcomes. However, the chemokines and their receptors that particularly stimulate tumorigenesis, including CCR7, CXCL1, CXCL8, and CXCL12/CXCR4, could, consequently, act as poor prognostic markers for cancer patients.

Despite the substantial advances in our understanding of the complex nature of the chemokine system in tumor biology, knowledge about the multifaceted roles of chemokines and their prognostic value in different types of cancers, especially in response to diverse anticancer therapies, is still limited. Nonetheless, a considerable number of chemokine receptor inhibitors targeting different chemokine signalling pathways are currently being evaluated in many preclinical studies and clinical trials, with encouraging results when used in combination with chemotherapy or immune checkpoint therapy. For the validation of specific chemokines and their receptors as prognostic markers of specific cancer types, further extensive studies are essential to decipher their distinct roles and the action mechanisms involved in cancer progression.

## Figures and Tables

**Table 1:** Chemokine receptors with their ligand pairings in humans and mice and various kinds of immune cells expressing chemokine receptors [8,13,14,15].

No.	Chemokine Receptors	Ligands ^a^	Immune Cells Expressing Chemokine Receptors
1	CCR1	CCL3, CCL4, CCL5, CCL6, CCL7, CCL8, CCL9, CCL13, CCL14, CCL15, CCL16, CCL23	T_h_1, T_h_2, T_h_9, T_h_17, T_RM_ cells, DCs, neutrophils, macrophages, monocytes, basophils
2	CCR2	CCL2, CCL7, CCL8, CCL12, CCL13, CCL16	T_h_1, T_h_17, T_reg_, NK cells, iDCs, neutrophils, monocytes, macrophages, MDSCs, basophils, platelets
3	CCR3	CCL4, CCL5, CCL6, CCL7, CCL8, CCL9, CCL11, CCL13, CCL15, CCL16, CCL23, CCL24, CCL26, CCL28	T_h_1, T_h_2, T_h_9, T_reg_ cells, neutrophils, macrophages, MDSCs, basophils, platelets, eosinophils, mast cells
4	CCR4	CCL3, CCL5, CCL17, CCL22	CD8^+^ T, T_h_2, T_h_17, T_h_22, T_reg_, skin- and lung-homing T, B cells, iDCs, monocytes, basophils, platelets
5	CCR5	CCL2, CCL3, CCL4, CCL5, CCL8, CCL11, CCL13, CCL14, CCL16	CD8^+^ T, T_h_1, T_h_9, T_h_17, T_reg_, T_EM_, T_RM_, NK cells, DCs, neutrophils, macrophages, monocytes
6	CCR6	CCL20	T_h_9, T_h_17, T_h_22, T_reg_, T_FH_, γδT, NK, NKT, B cells, iDCs, iLC
7	CCR7	CCL19, CCL21	Activated T, T_h_22, T_reg_, T_CM_, T_N_, T_RCM_, B cells, mDC
8	CCR8	CCL1, CCL4, CCL8, CCL16, CCL17, CCL18	T_h_2, T_reg_, skin T_RM_, γδT cells, macrophages, monocytes
9	CCR9	CCL25	T_h_17, T_h_22, gut-homing T, B cells, DCs, pDCs, IgA^+^ plasma cells, thymocytes
10	CCR10	CCL27, CCL28	T_h_17, T_h_22, skin homing T cell, T_reg_ cells, macrophages, IgA^+^ plasma cells
11	CXCR1	CXCL1, CXCL6, CXCL7, CXCL8	CD8^+^ T_EFF_, NK, neutrophils, macrophages, MDSCs, monocytes, basophils, mast cells
12	CXCR2	CXCL1, CXCL2, CXCL3, CXCL5, CXCL6, CXCL7, CXCL8	CD8^+^ T, NK cells, neutrophils, macrophages, MDSCs, monocytes, basophils, mast cells, platelets
13	CXCR3	CXCL4, CXCL9, CXCL10, CXCL11, CXCL13	CD8^+^ T_CM_, activated CD4^+^ T, T_h_1, T_h_9, T_h_11, T_reg_, T_FH_, T_EM_, NK, NKT, B cells, pDCs, platelets
14	CXCR4	CXCL12	Most T cells, T_reg_, B cells, iDCs, neutrophils, macrophages, MDSCs, monocytes, platelets, plasma cells, endothelial cells, precursors of endothelial cells
15	CXCR5	CXCL13	CD8^+^ T_EM_, T_h_17, T_CM_, T_FH_, T_FR_, B cells
16	CXCR6	CXCL16	T_h_1, T_h_17, T_h_22, γδT, NKT, NK, iLC, plasma cells
17	?	CXCL14	DCs
18	?	CXCL15	
19	?	CXCL17	
20	CX3CR1	CX3CL1	T, NK cells, DCs, macrophages, monocytes, microglia
21	XCR1	XCL1, XCL2	DC, cross-presenting CD8^+^ DCs

DC, dendritic cell; iDC, immature DC; iLC, innate lymphoid cell; NK cell, natural killer cell; NKT cell, natural killer T cell; MDSC, myeloid-derived suppressor cell; mDC, mature dendritic cell; pDC, plasmacytoid DC; T_h_, T helper cell; T_CM_, central memory T cell; T_EFF_, effector T cell; T_EM_, effector memory T cell, T_FH_, follicular helper T cell; T_FR_, follicular regulatory T cell; T_N_, naïve T cell; T_RCM_, recirculating memory T cell; T_reg_, regulatory T cell; and T_RM_, tissue-resident memory T cell. ^a^, chemokine ligands with black, red, and blue colours represent chemokine–chemokine receptor interactions that occur in both mice and humans, only humans, and only mice, respectively. Question marks indicate that the respective chemokine receptors are currently unidentified.

**Table 2:** Roles of chemokine system as prognostic factors in cancers.

Chemokines/Receptors	Cancer Types	Sites of Expression	Study Types	References
**Good prognostic markers**				
CCL2	Non-small cell lung cancer (NSCLC)	Tissue	Retrospective	[153]
CCL14	Hepatocellular carcinoma (HCC)	Tissue	Retrospective	[105]
CXCL10	Esophageal squamous cell carcinoma	Tissue	Prospective	[195]
CX3CL1/CX3CR1	HCC	Tissue	Prospective	[211]
CX3CL1	Gastric adenocarcinoma	Tissue	Prospective	[210]
XCL1	Acute lymphoblastic leukemia	Serum	Prospective	[216]
**Poor prognostic markers**				
CCL2/CCR2	Diffuse large B cell lymphoma (DLBCL)	Tissue	Prospective	[160]
CCL5	Breast cancer (BC)	Serum	Prospective	[81]
	Stage II BC	Tissue	Prospective	[162]
CCL20	Colorectal cancer (CRC)	Tissue	Prospective	[167]
CCL20/CCR6	HCC	Tissue	Prospective	[169]
CCL21/CCR7	Colorectal liver metastasis	Tissue	Prospective	[169]
CCR7	Solid tumors	Tissue	Meta-analysis	[178]
CXCL1	Various cancers	Tissue, urine, serum	Meta-analysis	[181]
CXCL8	CRC	Tissue	Prospective	[167]
	CRC	Serum	Prospective	[188]
	Cervical Cancer	Tissue	Prospective	[189]
	Lung adenocarcinoma	Tissue	Prospective	[190]
CXCL10	DLBCL	Serum	Prospective-retrospective	[196]
CXCL12	Esophageal cancer	Tissue	Prospective	[202]
CXCR4	Acute myelogenous leukemia (AML)	AML cells	Prospective	[203]
	Early BC	Tissue	Prospective-retrospective	[205]
	BC	Tissue	Meta-analysis	[204]
	HCC	Circulation and/or tissues	Meta-analysis	[206]
	NSCLC	Tissue	Meta-analysis	[207]
CX3CL1/CX3CR1	Pancreatic ductal adenocarcinoma	Tissue	Retrospective	[213]

For detailed information, please see the text.
